# Sequencing and Analysis of *Wolbachia* Strains from A and B Supergroups Detected in Sylvatic Mosquitoes from Brazil

**DOI:** 10.3390/microorganisms12112206

**Published:** 2024-10-31

**Authors:** Luísa Maria Inácio da Silva, José Irnaldo da Silva, Alexandre Freitas da Silva, Filipe Zimmer Dezordi, Lais Ceschini Machado, Si Qin, Hang Fan, Yigang Tong, Túlio de Lima Campos, Marcelo Henrique Santos Paiva, Gabriel Luz Wallau

**Affiliations:** 1 Departamento de Entomologia, Instituto Aggeu Magalhães, Fundação Oswaldo Cruz, Recife 21040-900, PE, Brazil; inacio.m.luisa@gmail.com (L.M.I.d.S.); joseirnaldosilva@gmail.com (J.I.d.S.); alexfreitasbiotec@gmail.com (A.F.d.S.); zimmer.filipe@gmail.com (F.Z.D.); laisceschini@gmail.com (L.C.M.); marcelo.paiva@fiocruz.br (M.H.S.P.); 2 Núcleo de Bioinformática e Departamento de Entomologia, Fundação Oswaldo Cruz, Recife 21040-900, PE, Brazil; tulio.campos@fiocruz.br; 3 State Key Laboratory of Pathogen and Biosecurity, Beijing Institute of Microbiology and Epidemiology, Beijing 100071, China; siqin346@163.com (S.Q.); siqin345@163.com (H.F.); 4 Beijing Advanced Innovation Center for Soft Matter Science and Engineering (BAIC-SM), College of Life Science and Technology, Beijing University of Chemical Technology, Beijing 100029, China; tong.yigang@gmail.com; 5 Núcleo de Ciências da Vida, Centro Acadêmico do Agreste, Universidade Federal de Pernambuco (UFPE), Caruaru 50670-901, PE, Brazil; 6 Department of Arbovirology and Entomology, Bernhard Nocht Institute for Tropical Medicine, WHO Collaborating Center for Arbovirus and Hemorrhagic Fever Reference and Research, National Reference Center for Tropical Infectious Diseases, Bernhard-Nocht-Straße, 74, 20359 Hamburg, Germany; 7 Universidade Federal Santa Maria (UFSM), Santa Maria 97105-900, RS, Brazil

**Keywords:** genomics, symbionts, horizontal transfer

## Abstract

*Wolbachia* are endosymbiotic bacteria that infect a wide range of arthropods and filarial nematodes, often manipulating host reproduction. The efficacy of *Wolbachia*-based interventions for dengue and chikungunya control has been validated through numerous field studies in recent years. This study aimed to investigate the diversity and prevalence of *Wolbachia* infections in sylvatic mosquitoes from two locations in Recife, Brazil. Multiple mosquito species were screened for *Wolbachia* using both target marker gene amplification coupled with Sanger sequencing and whole-genome sequencing (WGS) approaches. Phylogenetic analyses were conducted to classify *Wolbachia* strains into supergroups and assess their evolutionary relationships. Results revealed the presence of *Wolbachia* in eleven mosquito species examined, with different infection rates. Both supergroups A and B of *Wolbachia* strains were identified, with *Aedes albopictus* showing co-infection by both supergroups through the WGS approach. We also detected indirect evidence of *Wolbachia* horizontal transmission among mosquitoes and other distant host orders. This study provides valuable insights into the distribution and diversity of *Wolbachia* in sylvatic mosquitoes from Brazil and adds new important data about *Wolbachia* detection through target marker gene amplicon coupled with Sanger sequencing and WGS methods, highlighting its complementarity to ascertain the presence of *Wolbachia* in mosquito samples.

## 1. Introduction

During their lifespan, organisms undergo an intricate network of ecological interactions with other species and communities. These relationships span from mutually beneficial partnerships to fierce competition [1]. Notably, most multicellular eukaryotes harbor a diverse community of microbes, forming an intimate symbiotic microbiota, alongside occasional encounters with pathogenic invaders [2]. One of the most well-known arthropod symbionts are bacterial species from the *Wolbachia* genus. These bacteria belong to the order Rickettsiales and were first identified in 1923 in *Culex pipiens* ovaries [3]. It is estimated that *Wolbachia* naturally occurs in approximately 66% of all insect species and nearly 100% of filarial nematodes [4], inducing a variety of phenotypes with beneficial, neutral, or detrimental fitness impact to the host such as feminization, cytoplasmic incompatibility, and partenogenesis [5,6]. *Wolbachia pipientis* is the only species of a large and diverse genus which is subdivided in at least 17 supergroups (A–F, H–Q, and S) and further into strains that have diverged over hundreds of millions of years [7].

*Wolbachia* is known for its capacity to infect the host reproductive host cells and then undergo maternal vertical transmission to the next host generation [8]. This particular characteristic has been exploited as a biotechnological tool, in which two approaches of controlling insect population and arthropod-borne viruses using specific strains of the bacteria have been proposed: I—the reduction of vector population size based on cytoplasmic incompatibility, and II—the replacement of a mosquito population with a *Wolbachia*-infected arbovirus-refractory population [9,10,11]. The refractory strategy is based on the known phenotype of reduction or the blocking of arboviruses dengue virus (DENV), Zika (ZIKV), chikungunya (CHIKV), yellow fever (YFV), and West Nile (WNV) replication induced by the *w*Mel *Wolbachia* strain in *Aedes aegypti* and the *w*AlbB strain infection in Malaysian populations of the same species [12,13,14,15,16]. The establishment of *Wolbachia w*Mel populations in the field reduced 77% of dengue cases in Indonesia [17]. However, the success of the *Wolbachia* invasion of natural populations and these control programs depends on the behavior, biology, distribution, and frequency of *Wolbachia* in natural mosquito populations [18] as well as the biological features of the mosquito species [19]. Mosquitoes are a large group of insects from the Culicidae family comprising around 3.567 species classified into two subfamilies (Anophelinae and Culicinae) and 41 genera (https://mosquito-taxonomic-inventory.myspecies.info accessed on 30 October 2024). Until 2021, screening efforts for *Wolbachia* infection encompassed 217 species of Culicidae distributed across 22 different genera, representing approximately 6% of the total mosquito species documented [4]. Consequently, the current understanding of *Wolbachia* lineages infecting mosquito species is predominantly focused on the well-studied arbovirus vectors *Ae. aegypti*, *Ae. albopictus*, and *Cx. quinquefasciatus*. Knowledge gaps persist in the understanding of the diversity and prevalence of *Wolbachia* lineages in other mosquito species, particularly in Brazil and the Americas, highlighting the need for expanded research efforts in this field [4].

Despite recent rapid advancements in new molecular technologies that allows a higher resolution for the characterization of symbionts and their hosts, identifying and characterizing *Wolbachia* within infected hosts in natural settings remains challenging due to the limitations in detecting low *Wolbachia* concentration, the multiple sources of environmental contamination, the bacterium’s tissue tropism, and the difficulty in distinguishing between an active *Wolbachia* infection and integrated *Wolbachia* DNA in the host genome [20]. The detection and systematics of *Wolbachia* strains are primarily based on recovering molecular data that are commonly utilized for strain discrimination at various levels. These include highly sensitive methods based on PCR targeting genes as the *16S rRNA* gene, five multilocus sequence typing loci (MLST), *gatB*, *coxA*, *hcpA*, *fbpA*, and *ftsZ* genes, and the *Wolbachia* surface protein gene (*wsp*). To overcome these limitations and avoid false-negative results, more sensitive detection techniques are required.

In the present study, we detected and identified *Wolbachia* in sylvatic mosquitoes from Brazil using two different approaches: detection through PCR *wsp* gene amplification and Sanger sequencing, and whole-genome sequencing using the Illumina platform. Based on the phylogenetic reconstruction of the molecular data recovered, we further investigated supergroup classification and the assessment of possible horizontal transmission (HT), providing new insights into the interaction between *Wolbachia* and sylvatic mosquitoes.

## 2. Results

### 2.1. Wolbachia Screening in Mosquito Species

**Figure 1:**
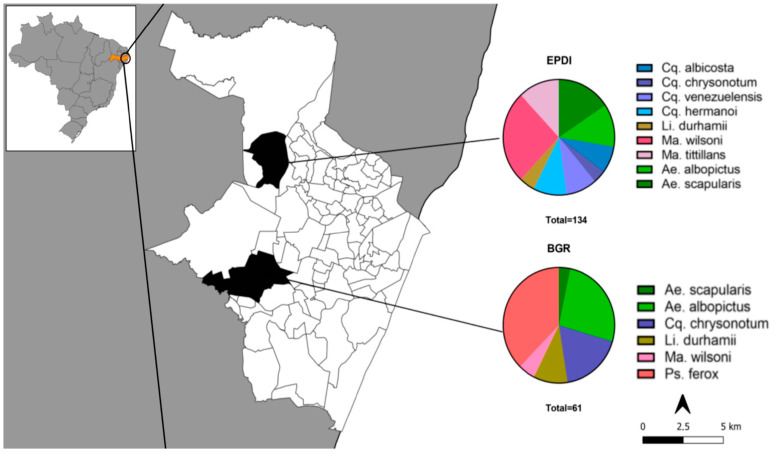
Map of collection sites and proportion of collected mosquito species in Recife Metropolitan region, Brazil. EPDI: Ecological Park of Dois Irmãos; BGR: Botanic Garden of Recife.

From January 2018 to December 2019, 195 samples belonging to 10 mosquito species were collected in the Ecological Park of Dois Irmãos (EPDI) and the Botanical Garden of Recife (BGR). These samples included five genera: *Aedes*, *Coquillettidia*, *Limatus*, *Mansonia,* and *Psorophora* (Table 1). One hundred ninety-five specimens were used for *Wolbachia* screening (Figure 1). All specimens analyzed were confirmed by COI barcoding, and all the obtained sequences were uploaded to the BOLD systems repository (https://boldsystems.org/index.php accessed on 30 October 2024) (Supplementary Table S1).

**Figure 2:**
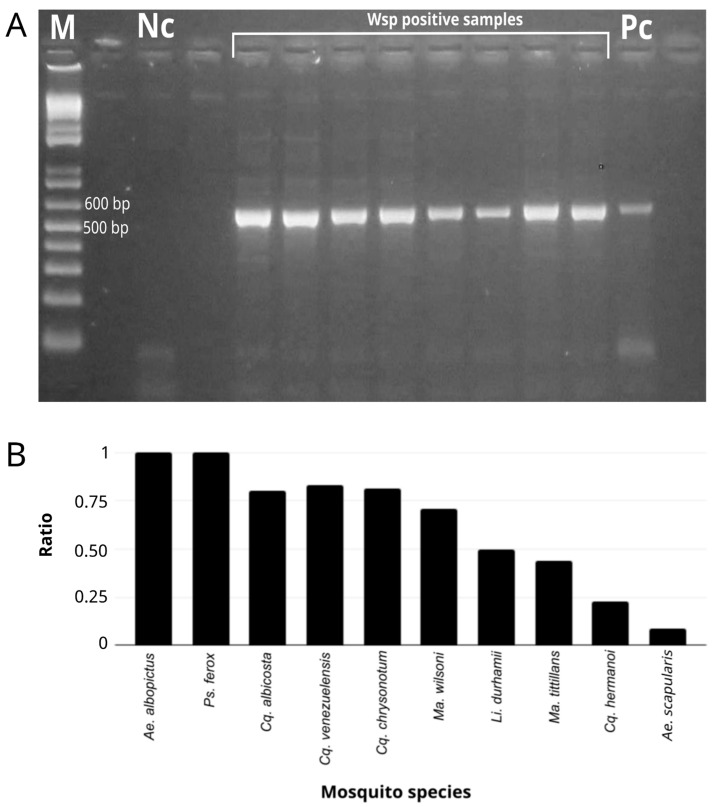
(**A**) Electrophoresis gel showing amplification of *wsp* gene in sylvatic mosquito samples. M: Marker (1 Kb Plus DNA ladder Thermo Fisher Scientific^®^, Waltham, MA, USA); Nc: negative control; Pc: positive control. PCR products were electrophoresed in 1.5% agarose gel, stained with ethidium bromide and visualized under UV light. (**B**) Ratio between mosquito samples tested and positive samples for *wsp* gene PCR amplification, per mosquito species (See Table 1).

Of the 195 samples tested for *Wolbachia*, 131 (67%) were positive by the PCR of the *wsp* gene, showing the expected amplicon size (Figure 2A). Samples that did not show amplification fragments within the expected size range (500 to 600 bp) were discarded (see Methods). Within the species analyzed, *Ae. albopictus* and *Ps. ferox* showed a higher positivity ratio for the *wsp* gene, while *Ae. scapularis* was the lowest (Figure 2B).

### 2.2. Wolbachia Sequencing

We obtained a total of 43 *wsp* sequences using the Sanger methodology (Table 2). These sequences were deposited to the GenBank under accession numbers OQ803315-OQ803353. There was no success in sequencing the *wsp* fragment from *Ae. scapularis* (two positive samples) and *Ps. ferox* (23 positive samples) specimens despite the high concentration and quality of fragments generated by *wsp* PCR (medium concentration between samples of 10 ng/uL for *Ps. ferox* and 18 ng/uL of *Ae. scapularis*).

Illumina sequencing generated a total of 1.5 billion paired-end raw reads, with read counts per pool ranging from 69.75 million for *Cx. quinquefasciatus* to 413.63 million for *Cq. hermanoi* (Table 3). After quality filtering and de novo assembly, these reads were assembled into approximately 1.3 million contigs. These contigs were then compared against the RefSeq genomes of *Wolbachia* from public databases. Following this comparative analysis, only 2804 contigs showing significant similarity to *Wolbachia* genomes were retained for further analysis.

**Figure 3:**
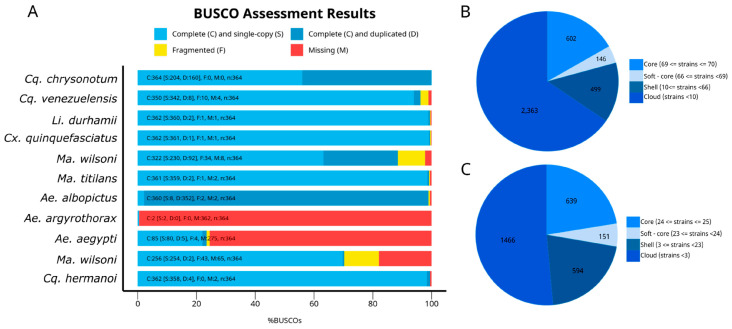
(**A**) BUSCO assessment of genome completeness in sylvatic mosquitoes from Brazil. (**B**) Distribution of genes within the genome of *Wolbachia* supergroup A. (**C**) Distribution of genes within the genome of *Wolbachia* and supergroup B.

Draft genome completeness was assessed using the BUSCO pipeline with the rickettsiales_odb10 database (Figure 3). This analysis revealed a high number of duplicated genes in *Ae. albopictus*. BUSCO analysis revealed that 10 out of the 11 species investigated showed Rickettsiales genes. We next sought to evaluate if these genes correspond to *Wolbachia*-specific genes by searching for the MLST and *wsp* genes. We were able to identify these genes in nine out of the ten species; only the *Ae. aegypti* dataset was negative for these genes indicating the absence of *Wolbachia* infection. Negative samples for these genes were excluded from further analyses.

**Figure 4:**
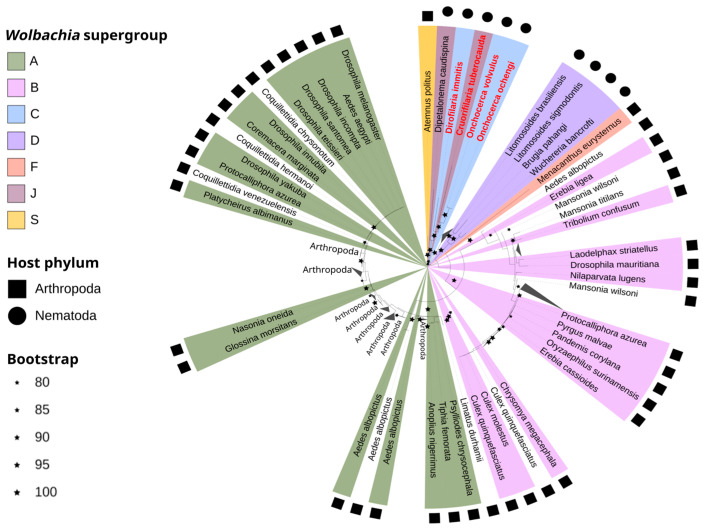
Maximum likelihood MLST genes *Wolbachia* phylogeny obtained in this study and databanks. Sequences generated in this project have a white background. Sequences from the databanks are colored according to the annotated supergroup. Sequences written in red are those whose basal node was used to root the tree. Black squares and circles represent the host phylum. Black stars represent a range of bootstraps values. Gray triangles represent collapsed clades, whose phylum of these sequences is noted in black.

After conducting BUSCO analyses, we performed a core genome analysis with supergroup A and B using Roary (Figure 4). Both supergroups A and B displayed a significant proportion of genes in the “Shell” category (499 and 594 genes, respectively), indicating a high degree of inter-group variability within these strains. Supergroup A has a slightly higher proportion of genes in the “Soft-core” category (present in 66–69 strains) compared to supergroup B (23–24 strains), suggesting the presence of a larger set of genes with intermediate conservation in supergroup A. Conversely, supergroup B exhibited a higher proportion of genes in the “Core” category (639 genes) compared to supergroup A (602 genes). The presence of a substantial proportion of genes in the “Cloud” category (genes present in less than 10 strains) in both supergroups (A and B) highlights the presence of numerous strain-specific genes.

### 2.3. Wolbachia Supergroup Classification Through Phylogenetic Analysis

For the phylogenetic analysis, 173 RefSeq genomes were obtained from the GenBank. After eliminating identical sequences from the same host species, 166 genomes remained.

To reconstruct the *wsp* phylogenetic tree, we combined the 43 *Wolbachia wsp* sequences generated from Sanger sequencing in this study with the dataset built, resulting in a total of 216 sequences for analysis.

All sequences obtained in this study clustered with high branch support (ultrafast bootstrap higher than 80) with supergroups A and B (Supplementary Figure S1). Sequences from *Ma. wilsoni*, *Ma. titillans*, and *Ae. albopictus* clustered within supergroup B, while the sequences from the other fives species analyzed clustered within supergroup A. These findings highlight the diversity of *Wolbachia* strains present in local mosquito populations and their association with different supergroups.

The *Wolbachia wsp* gene phylogeny, reconstructed from WGS data, positioned nine assembled genomes from this study and included other sequences from public databases, totalizing 184 sequences. It revealed the presence of two distinct *Wolbachia* supergroups (A and B) among the analyzed mosquito samples. Notably, *Coquillettidia venezuelensis*, *Coquillettidia chrysonotum*, *Coquillettidia hermanoi*, and *Limatus durhamii* harbored *Wolbachia* strains belonging to supergroup A, and *wsp* from the *Wolbachia* of *Li. durhamii* was grouped into supergroup A but close to the *wsp* samples from *Wu. bancrofti* (Supplementary Figure S2). As expected, *Aedes albopictus* individuals were found to be co-infected with *Wolbachia* strains from both supergroups A and B [21,22], suggesting a potential for complex interactions between these endosymbionts within this mosquito species.

**Figure 5:**
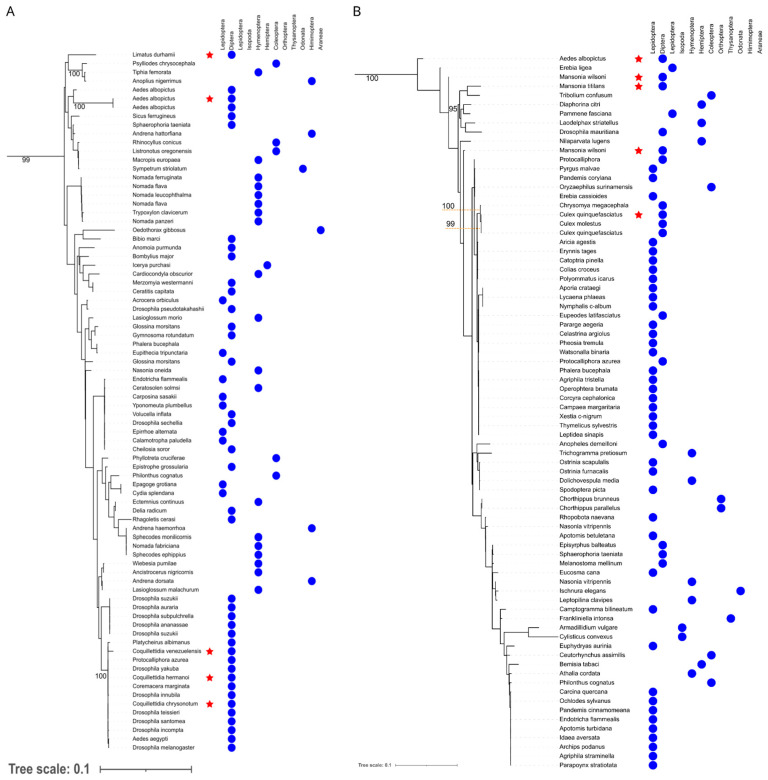
(**A**) Maximum likelihood phylogenetic tree of MLST genes supergroup A from *Wolbachia* species obtained in this study. Sequences generated in this project have a red star. (**B**) Maximum likelihood phylogenetic tree of MLST genes supergroup B from *Wolbachia* species obtained in this study. Sequences generated in this project have a red star. Bootstraps values from clades without sequences from this work and values <90 and were removed to improve visualization (see details in Supplementary Figure S3). Orange dashed lines indicate bootstrap values from the pointing clade. Red stars represent samples generated from this work. Both phylogenies are zoomed trees pruned from the complete MLST tree depicted in Figure 4.

The MLST phylogeny revealed similar patterns of *Wolbachia* strain distribution among the studied mosquito species. *Wolbachia* strains sequenced from *Cq. chrysonotum*, *Cq. hermanoi*, *Cq. Venezuelensis*, and *Li. durhamii*, clustered within supergroup A (Figure 4), while those from *Ma. wilsoni*, *Cx. quinquefasciatus*, *Ma. titillans*, and *Ma. wilsoni* clustered within supergroup B (Figure 5). As expected, *Ae. albopictus* harbored strains from both supergroups, indicating a potential co-infection (Supplementary Figure S3).

Within supergroup A (Figure 5A), a clear phylogenetic separation was observed between strains associated with arthropods and those associated with nematodes, suggesting host specialization. Notably, the *Wolbachia* strain found in *Cx. quinquefasciatus* from Recife clustered closely with other *Culex* mosquitoes (*Cx. quinquefasciatus* and *Cx. molestus*) from the database, highlighting the global distribution and prevalence of supergroup B (Figure 5B) *Wolbachia* within this species. Moreover, *Wolbachia* strains from supergroups A and B demonstrated a broad host range, infecting various arthropods, including mosquitoes, flies, and beetles. This suggests a propensity for horizontal gene transfer and adaptation to diverse hosts of *Wolbachia* from both supergroups [23].

All phylogenetic analyses consistently revealed the presence of *Wolbachia* supergroups A and B in the analyzed mosquito samples, underscoring their widespread distribution in the Recife city (Supplementary Data). Notably, *Ae. albopictus* exhibited co-infection with strains from both supergroups A and B in both WGS-based phylogenies (*wsp* and MLST), suggesting a complex symbiotic relationship within this species. However, the Sanger-based *wsp* phylogeny placed the *Ae. albopictus Wolbachia* strains exclusively within supergroup B, highlighting that potential methodological or sampling biases for *Wolbachia* detection must be taken into account. These differences may be attributed to several factors, including the higher resolution of WGS sequencing, higher resolution of the MLST analysis, and variations in species sampling among the three approaches.

### 2.4. Phylogenetic Evidence for Inter-Species Transmission of Wolbachia among Hosts

**Figure 6:**
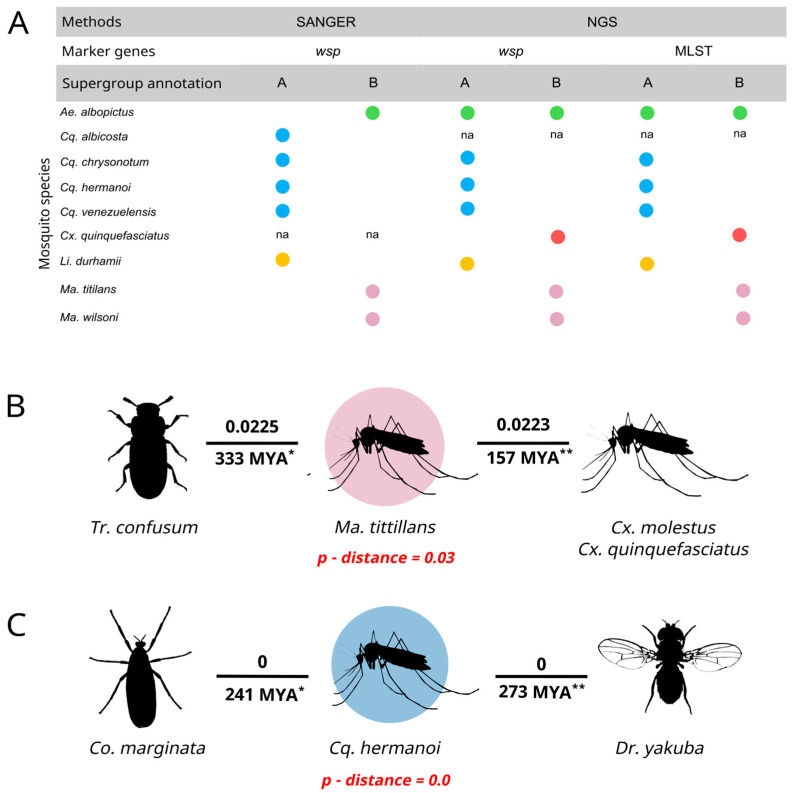
*Wolbachia* supergroups distribution per detection method and potential horizontal transfer events. (**A**) Comparison of *Wolbachia* mosquito sequence supergroups detected by the different approaches employed. (**B**) Schematic representative network based on the phylogenetic tree relations from Wolbachia MLST sequences of supergroup A found in mosquitoes and Wolbachia from other host species. (**C**) Schematic representative network based on the phylogenetic tree relations from Wolbachia MLST sequences of supergroup B found in mosquitoes and Wolbachia from other host species. Blue and pink circles represent *Mansonia* and *Coquillettidia* genera, respectively, being sequences from this work. Figures without circles are from the database. Numbers above the line represents the *p-distance* between *Wolbachia* sequences. Numbers under the line are the estimated divergence time in millions of years (MYA) between the hosts. The host species are the following: *Tribolium confusum*, *Coremacera marginata*, *Culex molestus*, *Culex quinquefasciatus,* and *Drosophila yakuba*. * Source: [24]; ** Source: [25] na: not analyzed. *p-distance*: average *p-distance* of differing nucleotide sites among all MLTS sequences found in the hosts.

Through the observation of the MLST phylogeny, we selected nine of our sequences that clustered together within the same clade with other *Wolbachia* strains detected from hosts other than mosquitoes. Distance matrix sequence analysis showed three of them that had high nucleotide identity (*p-distance* < 0.03) when compared to *Wolbachia* from mosquitoes (Figure 6B). Comparisons between *Cx. quinquefasciatus* and *Cx. molestus*, and *Cq. hermanoi*, *Co. marginata,* and *Dr. yakuba* showed 100% identity (Figure 6B). Distance matrix sequence analysis revealed a low overall mean pairwise nucleotide distance of 0.06 between all *Wolbachia* sequences, and within supergroups A and B, 0.03 and 0, respectively.

## 3. Discussion

*Wolbachia* has gained substantial attention in recent years for its exploitation as a biological control tool to reduce the transmission of mosquito-borne viruses such as DENV, ZIKV, and CHIKV [26,27,28,29,30]. It is estimated that systematic releases of *Ae. aegypti* transfected with *Wolbachia* in Indonesia reduced 77% of dengue hospitalizations [27]. Moreover, there are several initiatives exploring the potential of using *Wolbachia*-infected mosquitoes to suppress wild mosquito populations and reduce the transmission of mosquito-borne diseases [31,32]. While the release of *Wolbachia*-infected mosquitoes has proven effective in reducing arbovirus transmission in several studies, the dynamics of *Wolbachia* infection in naturally occurring mosquito populations remain poorly understood, with just 6% of Culicidae species investigated for this purpose so far. In order to provide basic information about *Wolbachia* natural diversity in sylvatic mosquito populations that can be used to explore new vector and arbovirus control approaches, we employed a targeted amplification of target genes coupled with Sanger sequencing and whole-genome sequencing (WGS) for *Wolbachia* detection and diversity analysis in nine sylvatic mosquitoes species and two laboratory raised species in Brazil, including epidemiologically important species such as *Cq. venezuelensis* and *Cq. crhysonotum* that may participate in arbovirus transmission cycles in Brazil [33].

In the present study, our results showed the presence of the *Wolbachia wsp* gene in 9 of the 11 evaluated species, with sequences belonging to supergroups A and B. This is the first record of *Wolbachia* in eight sylvatic mosquito species: *Ma. wilsoni*, *Cq. albicosta*, *Cq. venezuelensis*, *Cq. chrysonotum*, *Cq. hermanoi*, *Ae. scapularis*, *Li. Durhamii,* and *Ps. ferox.* One of the main differences in the results of applying amplicon coupled with Sanger sequencing and WGS approaches was the observation of the superinfection of *Ae. albopictus* with two strains of *Wolbachia* detected through genomic sequencing. *Wsp* and MLST markers recovered from WGS ascertained the presence of supergroups A and B, which differs from the result obtained by Sanger sequencing, which only allowed the detection of *Wolbachia* sequences from supergroup B. In this approach, *Wolbachia* was detected with PCR using specific *wsp* gene primers, which amplified strains from both supergroup A and supergroup B [34]. Differentiation between these supergroups is only achieved by Sanger sequencing. However, two main issues may arise with this: the presence of double peaks in the electropherogram or the amplification of only one strain [4,35,36]. The latter scenario may result from the preferential amplification of the more abundant strain, even in cases of superinfection. This likely explains the failure to detect lineage A using this method.

*Ae. albopictus* is the most investigated mosquito species for the presence of *Wolbachia* and there is a consensus in considering that this species is naturally infected with the presence of the bacteria with both *w*AlbA and *w*AlbB strains [4,37,38]. The differences regarding these *Wolbachia* strains are mainly related to its distribution at the *Ae. albopictus* population level. In the MLST phylogeny, the *Wolbachia* sequence from *Ae. albopictus* clustered together with sequences from a laboratory-maintained *Ae. albopictus* strain originating from Malaysia (GCF_024804185.1). This laboratory strain has a Malaysian nuclear genome background. Considered an invasive and anthropophilic species, *Ae. albopictus* is widely distributed in Brazil in forest regions and in sylvatic-urban interfaces, being considered an important bridge vector of viruses such as DENV, CHIKV, and YFV, but it was not yet implicated as a major arbovirus vector in Brazil [39,40,41].

Regarding the diversity of the hosts (mosquito species), the same mosquito genera were collected in both Atlantic Forest collection sites. At the same time, *wsp* amplification and Sanger sequencing resulted in *Wolbachia* sequences from *Li. durhamii* and *Cq. chrysonotum* from different collection sites clustering together. Two other studies detected *Wolbachia* in mosquitoes in Brazil: the first surveyed *Ae. albopictus* and the other *Cx. quinquefasciatus*, using the same Sanger methodology showing no variation in *wsp* presence within or among mosquito populations in all collection sites [42,43]. Another study in Brazil using the same detection approach observed 93% of mosquitoes collected species, with *Cx. quinquefasciatus*, *Ma. titilians*, *Ae. albopictus*, and *Li.* spp. positives [44].

Low diversity within *Wolbachia* genes and incongruences among Wolbachia’s host phylogenies are characteristics observed in several other studies [20,36,42,43,45,46]. Our phylogenetic analysis revealed unexpected clusters of *Wolbachia* sequences with high identity from phylogenetically distant hosts that diverged tens of millions of years ago. For example, the *Wolbachia* sequence from *Ma. tittilans* clustered with that of *Cx. molestus* from the database, with 99.9% identity, despite the divergence between their hosts of 157.02 million years ago [25]. Another example was observed between *Dr. yakuba* and *Cq. hermanoi*, with 100% identity between their *Wolbachia* sequences, despite the divergence between Drosophilidae and Culicidae estimated at approximately 273 million years ago [25]. Considering the five-gene cluster analysis (MLST), the known variability in *Wolbachia* evolutionary rates (ranging from 10 times than the mitochondrial genome, with some reaching nuclear rates) [20], and the already known horizontal transfer phenomenon of *Wolbachia*, the observed groupings suggest a possible HT event between these hosts. Once difficult to observe occurring in nature in real time, since *Wolbachia* HT occurs on generational and evolutionary time scales, incongruences in *Wolbachia* host phylogeny and low diversity are being used as evidence to support HT [23,45,47,48]. Our results are consistent with Gomes et al. (2022), who compared orthologous genes in distant *Wolbachia* hosts. They found that 6 of the 17 host species harboring *Wolbachia* supergroups A and B shared *Wolbachia* with over 95% similarity, suggesting recent and frequent HT. Notably, this occurred even among phylogenetically distant hosts like Hymenoptera, Coleoptera, and Diptera, including mosquitoes like *Ae. albopictus* [23].

However, not all cases of high identity between *Wolbachia* sequences are necessarily horizontal transmission events. The *Wolbachia* sequences of *Cx. pipiens molestus* and *Cx. quinquefasciatus* clustered together in MLST phylogeny and showed 100% identity. Both species belong to the *pipiens* complex and diverged about 13.5 million years ago [25,49]. In this case, the high identity can be explained by the recent divergence between the two species and the low rate of *Wolbachia* evolution in some lineages.

The initial sequencing of the *Wolbachia* genome (*w*Mel strain in *Drosophila melanogaster*, Supergroup A) revealed a compact genome of 1.3 Mb containing numerous mobile genetic elements [1,50]. Subsequent analyses have demonstrated that the *Wolbachia* pan genome comprises a core set of genes conserved across all strains and a variable accessory genome responsible for substantial size variation (1.2–1.8 Mb) [1,51]. Notably, strictly vertically-transmitted mutualistic *Wolbachia* in nematodes possess smaller genomes (0.96–1.1 Mb) compared to parasitic *Wolbachia* in arthropods, which exhibit occasional host-switching and acquire mobile elements such as bacteriophage WO, transposons, and plasmids [1]. Our genomic analysis, utilizing the rickettsiales_odb10 database for BUSCO analysis, revealed distinct patterns in *Aedes* mosquitoes from Recife. While *Ae. albopictus* displayed a high number of duplicated genes, potentially indicating co-infection or recombination events, *Ae. argyrothorax* and *Ae. aegypti* exhibited numerous missing genes. This included the absence of key *Wolbachia* marker genes such as *wsp* and the MLST genes, suggesting a likely absence of *Wolbachia* endosymbionts in these latter species. It is worth noting that the use of the rickettsiales_odb10 database may have led to the annotation of genes from other Rickettsiales bacteria, potentially contributing to the initial observation of “missing genes”. However, the targeted search for specific *Wolbachia* markers confirmed the absence of *Wolbachia* in *Ae. argyrothorax* and *Ae. aegypti*. Although some studies have reported the presence of *Wolbachia* naturally infecting *Aedes aegypti* in specific populations [36,52], a study analyzing *Aedes aegypti* from 27 countries found no evidence of natural *Wolbachia* infection [53].

The initial characterization of *Wolbachia* genetic diversity relied on *16S rRNA* and the *wsp* gene, but limitations in these markers led to the development of a multilocus sequence typing (MLST) system based on conserved housekeeping genes [35]. While the reliability of MLST has been questioned, with whole-genome sequencing proposed as a superior alternative [54], comparative genomic analysis indicates that MLST typing remains largely valid for both the supergroup identification and detection of closely related strains [1]. MLST-based relationships are largely consistent with genome-wide analysis, suggesting that MLST remains a valuable tool, particularly when whole-genome sequencing is not feasible [1]. In our MLST analysis, *Cq. chrysonotum*, *Cq. venezuelensis*, *Cq. hermanoi*, and *Li. durhamii* were grouped into supergroup A, while the remaining species were grouped into supergroup B. Additionally, the samples from *Ma. wilsoni* and *Ma. titillans* clustered together, while the sample from *Cx. quinquefasciatus* grouped with other *Culex* mosquito samples available from databases. Notably, a superinfection with the *Wolbachia* strains from both supergroups A and B was identified in *Ae. albopictus*. This finding, only possible through MLST analysis, underscores the importance of this method in revealing complex infection patterns that may have significant implications for mosquito biology and control. While the *wsp* gene is commonly used for differentiating *Wolbachia* strains, it may not be sufficient to identify co-infections, as demonstrated in our study.

In summary, advancements in target enrichment protocols and whole-genome typing methods, coupled with an improved set of MLST loci, are facilitating a more comprehensive understanding of *Wolbachia’s* genetic diversity and phylogenetic classification across diverse arthropod hosts and ecological niches [1]. However, the concordance between these approaches remains to be fully elucidated, particularly given the limitations of the current study, including the incomplete overlap of samples analyzed by both methods and the relatively small sample size. A more robust assessment with an increased sampling of mosquito species is warranted to comprehensively characterize the diversity and prevalence of *Wolbachia* within these populations. Such insights would be invaluable for informing strategies to manipulate *Wolbachia* infections in mosquitoes for arbovirus transmission control.

## 4. Conclusions

In this study, we aimed to investigate the *Wolbachia* natural diversity within mosquitoes in a sylvatic environment in Recife, Pernambuco-Brazil, highlighting both the potential and limitations of different detection and characterization methods. This is the first record of *Wolbachia* in eight sylvatic mosquito species: *Ma. wilsoni*, *Cq. albicosta*, *Cq. venezuelensis*, *Cq. chrysonotum*, *Cq. hermanoi*, *Ae. scapularis*, *Li. Durhamii,* and *Ps. ferox.*

We observed the co-infection of *Ae. albopictus* with multiple *Wolbachia* strains using whole-genome sequencing (WGS), which was not detected through Sanger sequencing alone. This underscores the enhanced sensitivity of genomic approaches for identifying more than one *Wolbachia* strain within a single host. We also observed close relationships between *Wolbachia* from mosquitoes and *Wolbachia* detected in other host orders, indicating a possible history of horizontal transmission between them. Both findings have important implications for understanding *Wolbachia*–host interactions and their potential impact in *Wolbachia* diversity.

## 5. Methods

### 5.1. Study Area and Mosquito Collection

Collection sites were distributed within the Recife municipality, capital of Pernambuco state, the Northeast region of Brazil. Recife is a seaside town and displays a year-long tropical climate, with a maximum mean temperature of 29 °C and minimum of 21.8 °C, and 77% relative air humidity. Sample collections were performed in two protected Atlantic Forest areas: the Ecological Park of Dois Irmãos (EPDI), which comprises an area of 384.42 hectares (−8.012915, −34.944996) and the Botanical Garden of Recife (BGR) with 11.23 hectares (−8.066140, −34.963022). The EPDI is also a zoological center and is located 10 km from BGR. The two places were chosen to maximize the sampling of the mosquito diversity from a sylvatic-urban interface.

Mosquito collections were performed using entomological nets. This instrument was chosen over other types of traps, since entomological aspirators are not specific for mosquitoes and capture many other organisms and plant remains from forested environments. Collections were carried out in each location once a month, from January 2018 to December 2019, between 9 a.m. and 12 a.m., and between 2 p.m. and 4 p.m., aiming to collect species with different daytime activities.

### 5.2. Mosquito Species Identification

The mosquito specimens were morphologically identified at the species/genus level based on the neotropical Culicidae keys from Lourenço-de-Oliveira [55] and Forattini [56], considering morphological characters present on the main body parts of adult female mosquitoes. Identified specimens were labeled and stored at −20 °C for later use.

All specimen manipulation was carried out on a cold surface (−20 °C) as swiftly as possible to prevent DNA/RNA degradation. Following taxonomic identification, specimens were dissected and separated into abdomen, head, legs, and thorax. The dissected abdomens were individually processed for DNA extraction following the protocol outlined by Ayres et al. (2003), and PCR reactions were performed to amplify the *Cytochrome oxidase subunit I* barcode (COI) to confirm the morphological species identification.

PCR for the COI gene was performed for each individual, following the protocol described in Paiva et al. [57].

### 5.3. Wolbachia Molecular Detection

All specimens were first screened individually for the presence or absence of *Wolbachia* through *wsp* PCR amplification. Primers and PCR reaction followed [34] protocol, which amplified a product ranging from 590 to 632 bp, varying based on the *Wolbachia* strain (Table 4).

In both COI and *wsp* reactions, we added a positive control that was extracted from *Ae. albopictus* previously caught in Recife, which naturally harbor groups A and B strains [43]. Both PCR products from *wsp* and from supergroup typing were visualized through 1.5% agarose gel in 0.5X TBE, stained with 10 mg/mL ethidium bromide, and visualized in an ultraviolet transilluminator.

### 5.4. Wsp and COI Sanger Sequencing and Quality Assessments

Sequencing of COI and *wsp* PCR-positive samples were performed in both strands on an ABI 3500xl sequencer (Applied Biosystems, Foster City, CA, USA) with the ABI PRISM BigDye Terminator Cycle Sequencing v 3.1 Ready Reaction kit (Applied Biosystems^®,^ Foster City, CA, USA). Forward and reverse sequences were analyzed using the program CodonCode Aligner (v.3.7.1) (CODONCODE, 2021), in which only sequences with a Phred value above 20 were used.

COI sequences for species molecular identification were used for a BLASTn analysis against the NCBI and BOLD systems [58] database (retrieved on 4 March 2020). We considered a species identification match when identities were between 98 and 100% within a coverage of 100% between sequences identified taxonomically in this work and sequences annotated in the database.

### 5.5. Wolbachia Genome Sequencing Through NGS and Quality Assessments

After molecular identification, a minimum of 5 and maximum of 22 specimens collected were grouped into pools for DNA extraction and library preparation. Samples of *Ae. aegypti* and *Ae. argyrothorax* were obtained from our existing database and included in this study. DNA libraries for *Cq. chrysonotum*, *Cq. venezuelensis*, *Cx. quinquefasciatus*, *Li. durhamii*, *Ma. wilsoni*, *Ma. titillans*, *Ae. argyrothorax*, *Ae. albopictus*, *Ae. argyrothorax*, and *Ae. aegypti*, were prepared using the Illumina DNA Prep kit (ILLUMINA, San Diego, CA, USA) according to the manufacturer’s instructions. We adopted a paired-end approach with 75 base pairs for each read, utilizing Reagent Kit V3 with 150 cycles. The libraries were sequenced on the Novaseq sequencing platforms (ILLUMINA, San Diego, CA, USA) at IAM/FIOCRUZ. Total DNA of *Ma. wilsoni* and *Cq. hermanoi* was sequenced using the HiSeq 2000 platform with a paired-end approach of 300 cycles (ILLUMINA, San Diego, CA, USA) in collaboration with the Beijing Institute of Microbiology and Epidemiology, Beijing, China.

MultiQC v1.10 [59] was utilized to streamline the comprehensive results from the extensive dataset generated by FastQC [60]. Following this, all raw reads were subjected to trimming using Trimmomatic tool version 0.3588 [61], aiming to eliminate adapter sequences and ensure a minimum sequence quality threshold of Phred score ≥ 20.

### 5.6. De Novo Assembly and Quality Assessment of Wolbachia Genomes

After quality analysis, for each isolate, the paired-end filtered data were used in *de novo* assembly. The assembly was performed using the MEGAHIT assembler version 1.2.9 [62]. To recover the *Wolbachia* contigs from the host genomic sequences, we performed sequence comparisons with GenBank records using the Basic Local Alignment Search Tool (BLAST) through BLASTn and tBLASTx [63]. These BLAST searches aimed to confirm the identity of the *Wolbachia* genome with sequences in the NCBI database. Only results with query coverage approaching 100%, a high identity exceeding 95%, and a bitscore greater than 500 were considered for further analysis.

The assessment of genome completeness was carried out utilizing the Benchmarking Universal Single-Copy Orthologs (BUSCO) pipeline [64] in conjunction with the rickettsiales_odb10 database. Prokka (Version 1.14.6) [65], a dedicated prokaryotic genome annotation pipeline, was employed to identify and annotate relevant features within assembled contigs. These features included coding sequences (CDSs) and signal leader peptides. This comprehensive approach enabled the annotation of essential genome features within contigs, CDSs, and completeness. Furthermore, we only included in the annotation analysis database-sourced *Wolbachia* genomes with at least 90% BUSCO completeness and at most 10% missing genes with the Rickettsiales_odb10 dataset.

### 5.7. Identification of Wolbachia Marker Genes

Following the assembly and filtering of *Wolbachia*-specific contigs, the sequences of marker genes *wsp* and Multilocus Sequence Typing System (MLST) (*gatB*, *coxA*, *hcpA*, *fbpA*, *ftsZ*) were identified individually using BLASTn and tBLASTx against a reference database. Upon identification and subsequent data filtering, the MLST marker gene sequences were concatenated using MEGA11 software [66] to facilitate downstream phylogenetic analysis. However, in the samples of *Ae. aegypti* and Ae. argyrothorax, an insufficient number of *Wolbachia* contigs were identified during the filtering process, and no *Wolbachia* MLST genes were detected. Consequently, these samples were excluded from further analysis.

### 5.8. Pangenomic Analysis of Wolbachia Supergroup A and B Using Roary

Following functional annotation with Prokka, the annotated genomes of supergroup A and B *Wolbachia* were used as input for pangenomic analysis using Roary software version 3.11.2 [67]. This analysis enabled the characterization of the core genome, comprising genes shared by most strains. The analysis generated two key outputs: a presence–absence matrix for accessory genes and a phylogenetic tree constructed based on this matrix. The roary_plot.py script was employed to visualize these results, generating a pie chart depicting the distribution of core and accessory genes, along with the phylogenetic tree reflecting the presence–absence matrix.

### 5.9. Construction of Wolbachia Databases for Evolutionary Analysis

To facilitate evolutionary analysis, we established individual databases for each output: *Wolbachia wsp* gene, MLST genes, and *Wolbachia* genomes. We retrieved *Wolbachia* genome sequences from the National Center for Biotechnology Information (NCBI) RefSeq database, accessing entries available up to October 2023. This dataset was filtered to include only complete genome assemblies and to exclude *Wolbachia* strains lacking host and supergroup information. After filtering, duplicated genomes were removed. Additionally, *wsp* and MLST gene sequences were obtained from the PubMLST database (https://pubmlst.org/), accessing entries available up to October 2023. Therefore, analysis was conducted using the constructed database and the samples generated in this work.

### 5.10. Maximum Likelihood Phylogenetic Analysis of Wolbachia Based on Wsp and MLST Markers

The *wsp* and MLST gene sequences obtained in this study were aligned with these sequences from the database using MAFFT (Version 7) with the auto strategy parameter [68]. Subsequently, these alignments were trimmed using Gblocks 0.91b [69] with default parameters to remove poorly aligned or divergent regions.

The resulting alignments were then used to construct maximum-likelihood trees, employing IQTREE (version 2.2.0) where branch support was assessed using the following options, each set to 1000 replicates: ultra-fast bootstrap, SH-aLRT support, local bootstrap support, and a Bayes Bayesian support [70].

For our phylogenetic analysis, two different markers were used: *wsp* and MLST. For Sanger, we used the *wsp* genes to reconstruct the phylogenetic tree. For WGS samples, we used both *wsp* and MLST genes. The resulting tree was then utilized as input for iTOL v6 [71], where visual and textual elements associated with the supergroup and host phylum were incorporated into the tree.

To further investigate the genetic distances among *Wolbachia* strains, we constructed a *p-distance* matrix using MEGA 11 software. P-distance quantifies the proportion of nucleotide sites that differ between two sequences [66]. We focused on the MLST genes of seven species that exhibited close phylogenetic relationships: *Ma. titilans*, *Tr. confusum*, *Cx. quinquefasciatus*, *Cx. molestus*, *Dr. yakuba*, *Cq. hermanoi*, and *Co. marginata*. We calculated both the overall mean *p-distance* and pairwise *p-distances* among these species.

## Figures and Tables

**Table 1:** Mosquitoes collected and analyzed for *Wolbachia* presence by PCR and sequencing. * Specimens were analyzed individually, ** *wsp* (+) is the number of samples where successful *wsp* PCR amplification was possible. Ratio was calculated by dividing the number of positive samples by the number of mosquitoes analyzed.

Genera	Species	Analyzed Samples *	PCR (*wsp* +) **	Ratio
*Aedes*	*Aedes (Stegomyia) albopictus* (Skuse, 1895)	32	32	1
	*Aedes (Ochlerotatus) scapularis* (Rondani, 1848)	23	2	0.08
*Coquillettidia*	*Coquillettidia (Rhynchotaenia) albicosta* (Chagas, 1908)	10	8	0.8
	*Coquillettidia (Rhynchotaenia) chrysonotum* (Peryassú, 1922)	16	13	0.8
	*Coquillettidia (Rhynchotaenia) hermanoi* (Lane and Coutinho, 1940)	13	3	0.23
	*Coquillettidia (Rhynchotaenia) venezuelensis* (Theobald, 1912)	12	10	0.83
*Limatus*	*Limatus durhamii* (Theobald, 1901)	12	6	0.5
*Mansonia*	*Mansonia (Mansonia) titillans* (Walker, 1848)	16	7	0.43
	*Mansonia (Mansonia) wilsoni* (Barreto and Coutinho, 1944)	38	27	0.71
*Psorophora*	*Psorophora (Janthinosoma) ferox* (von Humboldt, 1819)	23	23	1
Total		195	131	

**Table 2:** Number of sequences generated through Sanger approach by mosquito species.

Mosquito Species	Number of Sequences Generated Through Sanger Approach
*Aedes albopictus*	8
*Coquillettidia albicosta*	5
*Coquillettidia chrysonotum*	11
*Coquillettidia hermanoi*	1
*Coquillettidia venezuelensis*	5
*Limatus durhamii*	5
*Mansonia titillans*	3
*Mansonia wilsoni*	5

**Table 3:** Characteristics of *Wolbachia* genomes sequenced through NGS methodology from sylvatic mosquitoes of Brazil.

Species	*Wolbachia* PCR	Reads Before Trim	Reads After Trim	Total Contigs	Total *Wolbachia* Contigs	*Wolbachia* Total Bases	MMinimum Contig Length	AAverage CONTIG Length	MMaximum Contig Length
*Cq. chrysonotum*	Positive	131.55 M	127.79 M	173.734	274	2.015.132	298	7.354.5	106.288
*Cq. venezuelensis*	Positive	124.85 M	120.48 M	211.472	544	1.652.906	282	3.038.4	29.155
*Li. durhamii*	Positive	112.90 M	109.05 M	623.089	134	1.217.071	364	9.082.6	36.824
*Ma. wilsoni*	Positive	119.98 M	116.92 M	89.101	620	1.850.184	361	2.984.2	16.068
*Ma. titillans*	Positive	127.54 M	124.39 M	84.751	135	1.463.211	435	10.838.6	95.249
*Ae. albopictus*	Positive	95.49 M	91.48 M	383.575	258	2.301.836	281	8.921.8	57.926
*Ae. argyrothorax*	Not tested	105.92 M	101.66 M	26.609	11	25.108	387	2.282.5	6.763
*Cx. quinquefasciatus*	Not tested	69.75 M	67.54 M	66.247	147	1.275.966	282	8.68	87.362
*Ae. aegypti*	Not tested	113.00 M	108.99 M	19.868	16	1.065.177	1.616	66.573.6	267.577
*Ma. wilsoni*	Not tested	412.41 M	356.84 M	51.828	466	949.711	345	2.038	12.847
*Cq. hermanoi*	Not tested	413.63 M	344.45 M	188.535	211	1.501.655	282	7.116.8	41.774

**Table 4:** Primers used to screen for the presence of *Wolbachia* and to classify into supergroups.

Primer	Region	Sequence	Amplification Product Size (bp)	Reference
*Wsp*	*Wolbachia* surface protein: general for all strains	F: 5′-TGGTCCAATAAGTGATGAAGAAACTAGCTA-3′ R: 5′-AAAAATTAAACGCTACTCCAGCTTCTGCAC-3′	590–632	Zhou; Rousset; O’neil, 1998 [34]

## Data Availability

The data used in this manuscript can be found in the Supplementary Material.

## Supplementary Materials

The following supporting information can be downloaded at: https://www.mdpi.com/article/10.3390/microorganisms12112206/s1. Table S1: spreadsheet with records of *Wolbachia* and mosquitoes generated in this work and submitted to databank and its accession numbers. Table S2: spreadsheet with records of *Wolbachia* database used in this work. Figures S1–S3: Maximum likelihood *wsp* gene *Wolbachia* phylogeny obtained in this study and databanks using Sanger and WGS sequencing.
