# Supplementary figures and images for: Sequencing and Analysis of Wolbachia Strains from A and B Supergroups Detected in Sylvatic Mosquitoes from Brazil
Source: Microorganisms. 2024 Oct 31;12(11):2206. doi: 10.3390/microorganisms12112206 (PMC11596719; doi:10.3390/microorganisms12112206)

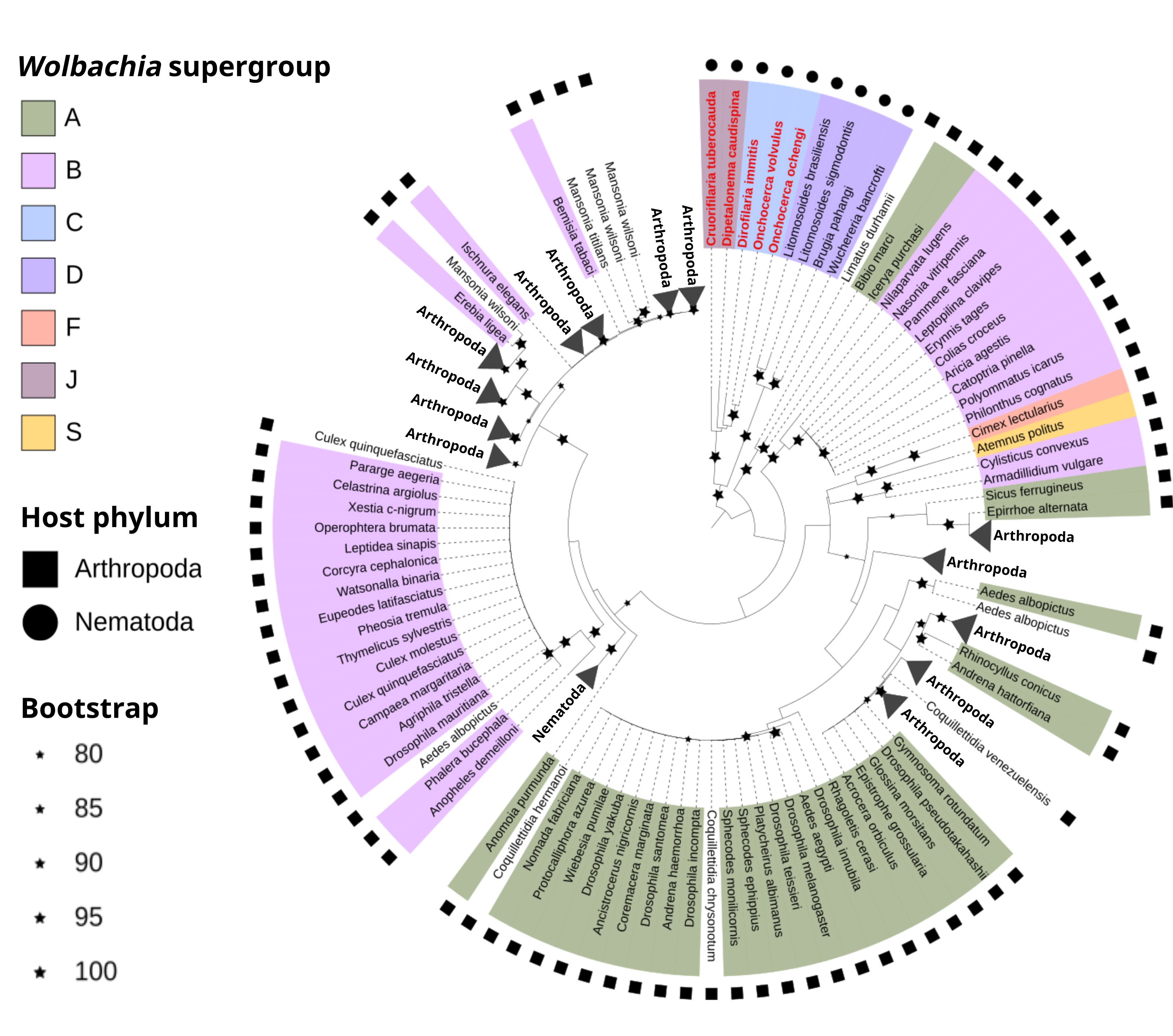

Supplement: Supplementary file 1 [file microorganisms-12-02206-s001.zip › Supplementary figure s2.png]

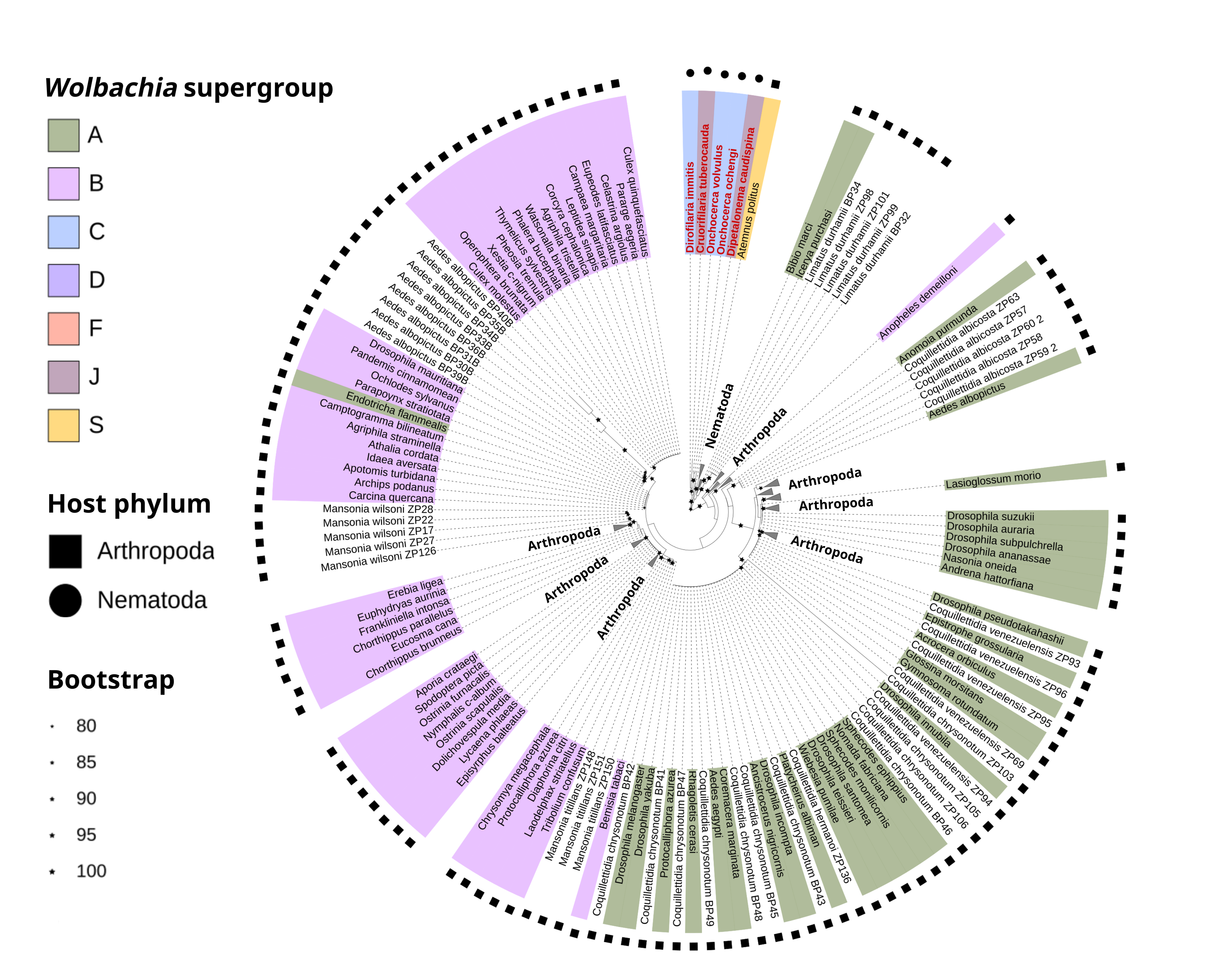

Supplement: Supplementary file 1 [file microorganisms-12-02206-s001.zip › Supplementary figure s1.png]

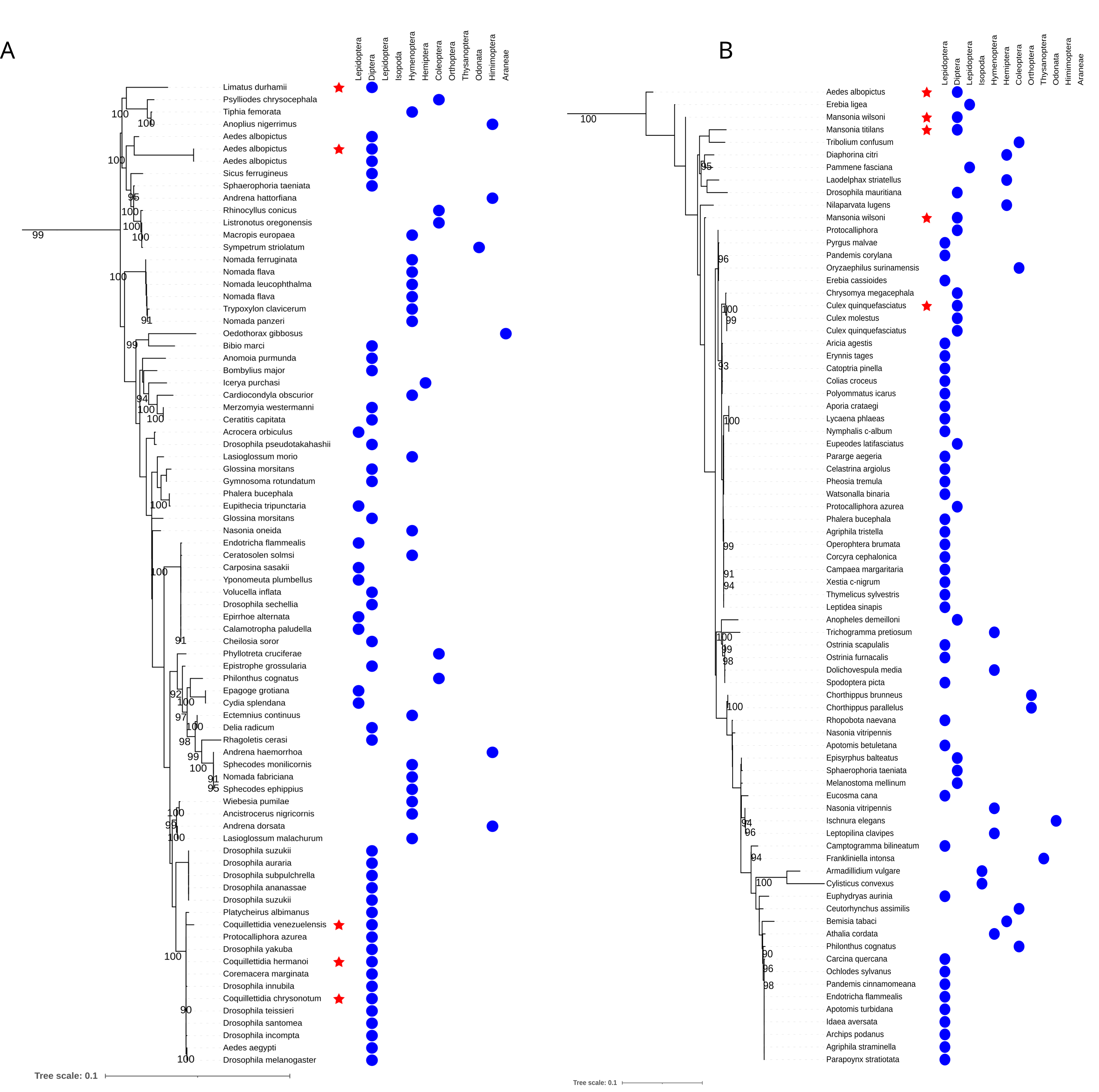

Supplement: Supplementary file 1 [file microorganisms-12-02206-s001.zip › Supplementary figure S3.png]
